# Linking histopathological changes in intervertebral disc with lumbar muscles strength

**DOI:** 10.6026/97320630019810

**Published:** 2023-07-31

**Authors:** Amlesh Kumar Yadav, Sridevi Nangali Srinivasa

**Affiliations:** Department of Anatomy, Sri Devaraj Urs Academy of Higher Education and Research, Kolar, Karnataka, India, 536101

**Keywords:** Degeneration, intervertebral disc, low back pain, histopathology

## Abstract

Low back pain (LBP) is a painful condition affecting 80% of word population at some point in their life. Lumbar intervertebral disc degeneration and reduced muscle force is one the major cause of LBP. Sixty-two patients with LBP undergoing discectomy were recruited after receiving consent. Lumbar muscles strength and VAS (visual analogue scale) for lumbar pain were checked, MRI scan were analysed. Histological degenerative changes were analysed. The study revealed histological degeneration at intervertebral disc has direct effect on increase in pain at lumbar region, decrease in strength of lumbar flexor and extensors muscles and degenerative changes seen on MRI scan.

## Background:

Low back pain (LBP) is a painful condition, localized below the costal margin and above the inferior gluteal fold, with or without referred pain to the lower limbs [1-2]. It is one of the global health problems, which adds to considerable socioeconomic burden affecting about 80% of world population at some point in their life [3-4]. It is the major cause of limited activity and absenteeism from work [5-6,7], resulting in a huge medical burden and economic cost [8-9]. According to the duration of low back pain, it can be classified as acute low back pain (ALBP) which persist for less than six weeks and chronic low back pain (CLBP) where the pain persists for more than three months duration of time [10], which is associated with histo-morphological, structural and atrophic changes and fatigability in para-spinal muscles [11-12,13]. Stability of the spine is maintained by three sub-units 1) the spinal column which includes intervertebral disc, facet joints, ligaments 2) the spinal muscles which includes flexor and extensor muscles group and 3) the neural control. A disturbance in the integrity and interplay between the stabilizing sub-units leads pathogenesis of low back pain [14- 15]. The specific cause of LBP is not yet understood but studies suggest that about 40% of the cause of LBP is because of the degenerative process of lumbar intervertebral disc (IVD) [16] and reduced cross-sectional area of core muscles is associated with reduced force generation leading to instability of spine, which is also one of the reasons for LBP [17-18]. Intervertebral discs (IVD) are fibro-cartilaginous, avascular, and anural structures which are located between the vertebral bodies and form the pathway for weight transmission from upper limb, head, neck, and thoracic spine to the lower limb [19]. IVD consists of superior and inferior end plates, outer annulus fibrosus (AF) and centrally placed nucleus pulposus (NP). Annulus fibrosus (AF) consists of fibroblasts like cells which synthesizes primary of type I collagen fibres which are present in the outer part and type II collagen present in the inner part [15] and are arranged concentrically at an angle of 300 on the transverse plane. Nucleus pulposus shows chondrocyte cells which synthesize mainly type II collagen fibres and are rich in extracellular matrix proteins mainly of hydrophilic proteoglycan (PG) [20]. Muscles surrounding lumbar spine (flexor and extensor) transmits most of the load from the entire body to the lower part of body, function as stabilizer of the spine and produces movements at the spine; as a result of which it plays a crucial role in functioning of spine. Studies suggest that muscle dysfunction, greater fat content and decreased muscle strength are common in LBP [21]. Therefore, it is of interest to histologically examine the lumbar IVD (intervertebral disc) and correlate histological changes with lumbar spinal muscles strength in patients with LBP (low back pain).

## Materials and methods:

This analytical study was conducted in Department of Anatomy in collaboration with Department of Radiology, Department of Orthopaedics and Department of Neurosurgery, at RL Jalappa Hospital and Research Centre, a teaching hospital of Sri Devaraj Urs Medical College, a constituent of Sri Devaraj Urs Academy of Higher Education and Research, Kolar, Karnataka, India.

## Sample size calculation:

Sample size calculation was done by using SPSS, version 22.0, with 90% power and 95% confidence interval [22]. A total of 62 subjects were enrolled in this study as case. The study has been approved by the central ethics committee, SDUAHER, KOLAR(R&I/90/2020-21) and written informed consent was obtained from subjects.

## Inclusion criteria:

Patients within the age group of 20-45 year, male and female with the history of low back pain, with or without radiating pain to the lower limb, having grade III, IV and V on pfirrmann grading on MRI scan was included in the study group

## Exclusion criteria:

Patients with cervical or thoracic intervertebral disc degeneration, malignancy, pregnancy, history of other joint pains, history of prior discectomy, smokers, patients having abdominal trauma, known case of rheumatoid arthritis or spinal infection were excluded from the study.

## Sample collection

62 patients who were recruited as study group which fulfils the inclusion criteria, after which consent from the patients was taken to enrol the patients for the study. Patients were than clinically examined, level of pain at lumbar region was recorded on VAS (visual analogue scale), muscle strength for the lumbar flexor and extensors muscles was recorded by manual muscle testing method, MRI scan was done and was graded according to pfirrmann grading system and lumbar intervertebral disc biopsy sample was collected during discectomy surgery which was stored in buffered 10% formaldehyde and was later processed for histopathological analysis.

## Pain assessment at lumbar region:

Patients recruited for the study was analysed for pain at the lumbar region by using Visual Analogue Scale (VAS). A Visual Analogue Scale (VAS) is one of the pain rating scales [23] the score is determined by measuring distance on 10 cm line, where 0 cm indicates no pain and 10 cm indicates maximum pain, intensity of pain increases as the distance increases from 0 cm to 10 cm (left to right).

## Manual muscle testing of lumbar spine muscles:

Patients included in the study group were tested for the muscle strength of lumbar spine (flexor and extensor muscles) by using manual muscle testing method.

## Lumbar Extensor muscles manual muscle testing:

## Position of the Patient:

Patient was asked to lie in prone position with hand clasped behind head and was instructed to raise head, shoulder, and chest off the table.

## Lumbar Flexor muscles manual muscle testing:

## Position of the patient:

Patient was asked to lie in supine position with hand clasped behind head and was instructed to raise head, Shoulder, and thoracic spine off the table.

## MRI scans analysis:

62 patients recruited in the study group were scanned for degenerative changes at lumbar intervertebral disc by MRI and were graded according to Pfirrmann grading system. The Pfirrmann grading system (Table 1) is widely used classification for intervertebral disc degeneration [24-25].

## Histological assessment of intervertebral disc:

Lumbar intervertebral disc biopsy sample were collected during discectomy surgery from the patients recruited as study group. Biopsy tissue was collected in 10% buffered formalin. Tissue sample were than processed by using tissue processing steps which includes fixation, dehydration and clearing, impregnation and embedding, paraffin block making, paraffin blocks were than sectioned at 4-6 µ m thickness by using microtome. Tissue sections were stained by using Haematoxylin and Eosin stain to identify chondrocyte cluster and granular changes in annulus fibrosus and nucleus pulposus, massion's tricrome stain to identify misalignment of the collagen fibres in annulus fibrosus and nucleus pulposus and alcian blue stain to identify mucous degeneration and histological variables were selected for further analyses [26] (Table 2).

## Statistical analysis:

Descriptive statistics such as frequency & proportion was calculated for categorical data and mean, standard deviation was calculated for continuous data. Chi square test was used to compare gender of study and control group, Paired t test was used to compare between group variable, p value <0.05 was considered as statistically significant.

## Results:

The patients with history of low back pain, within the age group of 20-45 years, both male and female undergoing for discectomy surgery after receiving consent from the patients were included in the study group. Our study revealed 58.06% were male and 41.64% were female among the patients and most prevalent age group was between 41-50 years with 59.68% followed by age group 31.40 with 32.26% patients respectively and the mean age of the patients was 40±5.75 respectively(Table 3) and mean of lumbar VAS was 5.88 among the study group. The MRI study revealed 41 patients with grade III pfirrmann degeneration and 21 patients with grade IV degeneration.

## Muscle strength of flexor and extensor muscles of lumbar spine:

Flexor and extensor muscles strength of lumbar spine were checked by manual muscle testing method before discectomy surgery in patients. Our study showed that there was no significant correlation between flexor and extensor muscle strength (Table 3). The study also revealed that there was no significant correlation between flexor strength and extensor strength.

## Histological characteristic of lumbar intervertebral disc:

**Figure 1:**
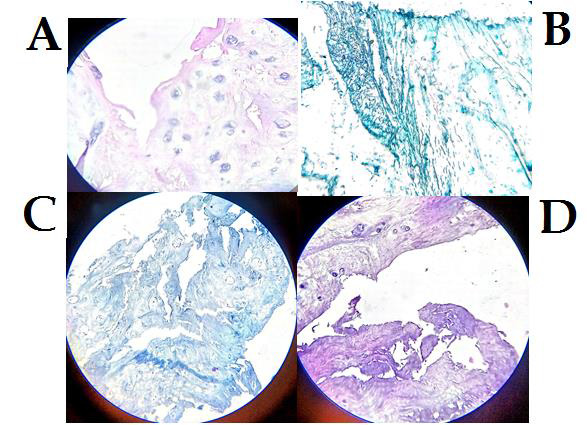
(A) H&E Stain shows chondrocyte cell in cluster; (B) Alcian Blue Stain shows mucous degeneration; (C) H&E stain shows granular changes; (D) Massion Trichrome stain shows concentric and radial tear in collagen fibers

Disc section from the patients undergoing surgery for back pain showed increased number and size of chondrocyte cells, which were rounded or elongated, with territorial matrix and chondrocyte cells were arranged in a group of two or three cells at places (Figure 1A). Presence of irregular areas with deposition of acid mucopolysaccharide which stained dark blue with Alcian blue suggesting mucous degeneration was observed in patient's disc section (Figure 1B). Ground substance appeared granular in patients (Figure 1C). Patient's disc section showed many tears in the annulus fibrosus interrupting the concentric arrangement of collagen fibres, these tears in annulus fibrosus could also be observed extending deep inside the nucleus pulposus (Figure 1D).

## Correlation between histopathological changes and flexor muscles strength:

The study revealed that there was highly significant correlation between flexor strength with mucous degeneration and granular changes with p value (<0.0001) and there was significant correlation with No. of cells, concentric tear & radial tear with flexor strength (p value 0.02) of study participants (Table 4).

## Correlation between histopathological changes and extensor muscles strength

Our study revealed that there was significant correlation between granular changes and mucous degeneration with extensor strength, also concentric tear showed significant correlation with extensor strength and no. of cells and radial tear showed no significant correlation with extensor strength (Table 5).

## Correlation between histopathological changes and pfirrmann grading:

The study revealed that there was statistically significant correlation between mucous degeneration and pfirrmann grades with p value (<0.0001) and other histological criteria were not showed significant correlation Pfirrmann (Table 6).

## Correlation between histopathological changes and VAS:

The study revealed that there was statistically significant correlation between mucous degeneration and concentric tear with visual analogous score and rest of histological criteria showed no significant correlation with visual analogous score (Table 7).

## Discussion:

Our study is first of its kind as per our knowledge; where histopathological changes was correlated with muscle strength of lumbar spine in patients with clinical history of low back pain and undergoing discectomy surgery. This study examined whether lumbar MRI, lumbar VAS, lumbar muscles strength measured by manual muscle testing method in routine clinical examination in patients with low back pain undergoing disectomy surgery for proplapsed disc is correlated with histopathological changes in lumbar intervertebral disc. Studies suggest that the main cause of intervertebral disc degeneration is loss of nucleus pulposus matrix, which in turn decreases the proteoglycan content and results in unequal transmission of force on intervertebral disc which marks the process of degeneration. In response to which chondrocyte cells proloferate to up-lift and compensate progressive extracellular loss [20]. In our study, we observed many chondrocyte cells with territorial matrix and proportion of cells clustered was similar in both AF and NP in prolapsed disc sample. Mucous degeneration, granular changes, concentric tears and radial tears all being prominently observed in histological section from the patients suggesting"degeneration through regeneration". Several studies have indicated that patients with LBP have significantly decreased abdominal muscle strength than asymptomatic patients [21-22,23]. In our study flexor muscles strength was significantly correlated with the histopathological change in lumbar intervertebral disc. Study suggests that size and quality of lumbar para-spinalis muscles were shown to be important factors for preventing relapse of LBP. Patients with chronic LBP tend to develop reduced lumbar muscle strength due to pain-induced movement reduction [24], which was evident in our study that extensor muscles strength of lumbar spine was significantly correlated with the histopathological changes occurring at lumbar intervertebral disc.

## Conclusion:

Results from our current study demonstrates that patients with low back pain undergoing discectomy surgery, showed degenerative changes such as increase in the number of chondrocytes, granular changes, mucous degeneration, concentric and radial tear; which showed significant correlation with lumbar spine flexor and extensor muscles strength, lumbar pain and degenerative changes on MRI scan. This suggests that histological degeneration at intervertebral disc has direct effect on increase in pain at lumbar region, decrease in strength of lumbar flexor and extensors muscles and degenerative changes seen on MRI scan.

## Figures and Tables

**Table 1:** Pfirrmann grading system

**Grade**	**Structural changes**
**Grade I**	[1] Disc is homogeneous with bright hyperintense white signal intensity and normal disc height
**Grade II**	[1] Disc is inhomogeneous but keeping the hyperintense white signal
	[2] Nucleus and annulus are clearly differentiated, and a gray horizontal band could be present
	[3] Disc height is normal
**Grade III**	[1] Disc is inhomogeneous with an intermittent gray signal intensity
	[2] Distinction between nucleus and annulus is unclear
	[3] Disc height is normal or slightly decreased
**Grade IV**	[1] Disc is inhomogeneous with a hypointense dark gray signal intensity
	[2] There is no more distinction between the nucleus and annulus
	[3] Disc height is slightly or moderately decreased
**Grade V**	[1] Disc is inhomogeneous with a hypointense black signal intensity
	[2] There is no more difference between the nucleus and annulus
	[3] The disc space is collapsed

**Table 2:** Variables of Histologic Assessment

**Histological parameters**	**Grades**
1. Cells (chondrocyte proliferation) Multiple chondrocytes growing in small rounded groups or clusters sharply demarcated by a rim of territorial matrix	0 = no proliferation; 1 = increased cell density; 2 = connection of two chondrocytes; 3 = small size clones (several chondrocytes, grouped together, 3-7 cells); 4 = moderate size clones (8-15 cells); 5 =huge clones (>15 cells)
2. Granular changes Amorphous granules within the fibrocartilage matrix	0 = absent; 1 = rarely present; 2 = present in intermediate amounts of 1 to 3; 3 =abundantly present
3. Mucous degeneration Irregular areas with an intense deposition of acid mucopolysaccharides staining dark blue with Alcian blue-PAS	0 = absent; 1 = rarely present; 2 = present in intermediate amounts of 1 to 3; 3 = abundantly present
4. Concentric tears Orientation of collagen fiber bundles in the annulus fibrosus	0 =absent; 1 = rarely present; 2 = present in intermediate amounts of 1 to 3; 3 = abundantly present
5. Radial tears Radiating defects extending from the nucleus pulposus to the outer annulus	0 =absent; 1 = rarely present; 2 = present in intermediate amounts of 1 to 3; 3 = abundantly present

**Table 3:** Comparison and significance level between flexor strength and extensor strength

	**extensor strength**	
	**Correlation coefficient**	**P value**
flexor strength	-0.197	0.12

**Table 4:** Correlation and significance level of histological parameters and flexor strength

**Histological parameters**	**Flexor strength**	
	**Correlation coefficient**	**P value**
No. of cells	0.306	0.02
Granular changes	0.49	<0.0001
Mucous Degeneration	0.776	<0.0001
Concentric tear	0.293	0.02
Radial tear	0.308	0.02

**Table 5:** Comparison and significance level of histological parameters and extensor strength

**Histological parameters**	**Extensor strength**	
	**Correlation coefficient**	**P value**
No. of cells	-0.149	0.25
Granular changes	0.387	<0.0001
Mucous Degeneration	0.597	<0.0001
Concentric tear	0.256	0.04
Radial tear	-0.08	0.952

**Table 6:** Comparison and significance level of histological parameters and Pfirrmann grades

**Histological parameters**	**Pfirrmann grades**	
	**Correlation coefficient**	**P value**
No. of cells	0.015	0.91
Granular changes	0.01	0.93
Mucous Degeneration	0.685	<0.0001
Concentric tear	-0.079	0.54
Radial tear	0.047	0.72

**Table 7:** Comparison and significance level of histological criteria and VAS

**Histological criteria**	**VAS**	
	**Correlation coefficient**	**P value**
No. of cells	-0.179	0.16
Granular changes	0.073	0.57
Mucous Degeneration	-0.342	0.007
Concentric tear	-0.335	0.008
Radial tear	0.141	0.28
